# Optimizing Nutritional Care with Machine Learning: Identifying Sarcopenia Risk Through Body Composition Parameters in Cancer Patients—Insights from the NUTritional and Sarcopenia RIsk SCREENing Project (NUTRISCREEN)

**DOI:** 10.3390/nu17081376

**Published:** 2025-04-18

**Authors:** Giuseppe Porciello, Teresa Di Lauro, Assunta Luongo, Sergio Coluccia, Melania Prete, Ludovica Abbadessa, Elisabetta Coppola, Annabella Di Martino, Anna Licia Mozzillo, Emanuela Racca, Arianna Piccirillo, Vittoria Di Giacomo, Martina Fontana, Maria D’Amico, Elvira Palumbo, Sara Vitale, Davide D’Errico, Valeria Turrà, Ileana Parascandolo, Tiziana Stallone, Livia S. A. Augustin, Anna Crispo, Egidio Celentano, Sandro Pignata

**Affiliations:** 1 Epidemiology and Biostatistics Unit, Istituto Nazionale Tumori IRCCS “Fondazione G. Pascale”, 80131 Naples, Italy; g.porciello@istitutotumori.na.it (G.P.); melania.prete@istitutotumori.na.it (M.P.); elvira.palumbo@istitutotumori.na.it (E.P.); sara.vitale@istitutotumori.na.it (S.V.); l.augustin@istitutotumori.na.it (L.S.A.A.); a.crispo@istitutotumori.na.it (A.C.); e.celentano@istitutotumori.na.it (E.C.); 2 Department of Urology and Gynecology, Istituto Nazionale Tumori IRCCS “Fondazione G. Pascale”, 80131 Naples, Italy; teresa.dilauro@istitutotumori.na.it (T.D.L.); elisabetta.coppola@istitutotumori.na.it (E.C.); davide.derrico@istitutotumori.na.it (D.D.); s.pignata@istitutotumori.na.it (S.P.); 3 Branch of Medical Statistics, Biometry and Epidemiology “G. A. Maccacaro”, Department of Clinical Sciences and Community Health, Dipartimento di Eccellenza 2023–2027, Università degli Studi di Milano, 20133 Milan, Italy; sergio.coluccia@unimi.it (S.C.); 4 Dietetics and Artificial Nutrition, Istituto Nazionale Tumori IRCCS “Fondazione G. Pascale”, 80131 Naples, Italy; ludovica.abbadessa@istitutotumori.na.it (L.A.); annabella.dimartino@istitutotumori.na.it (A.D.M.); vittoria.digiacomo@istitutotumori.na.it (V.D.G.); martina.fontana@istitutotumori.na.it (M.F.); v.turra@istitutotumori.na.it (V.T.); ileana.parascandolo@istitutotumori.na.it (I.P.); 5 Melanoma Cancer Immunotherapy and Innovative Therapy Unit, Istituto Nazionale Tumori IRCCS “Fondazione G. Pascale”, 80131 Naples, Italy; annalicia.mozzillo@istitutotumori.na.it (A.L.M.); 6 Experimental Clinical Abdominal Oncology Unit, Istituto Nazionale Tumori IRCCS “Fondazione G. Pascale”, 80131 Naples, Italy; emanuela.racca@istitutotumori.na.it (E.R.); 7 Otolaryngology and Maxillo-Facial Surgery Unit, Istituto Nazionale Tumori IRCCS “Fondazione G. Pascale”, 80131 Naples, Italy; arianna.piccirillo@istitutotumori.na.it (A.P.); 8 Colorectal Surgical Oncology Abdominal Oncology Department, Istituto Nazionale Tumori IRCCS “Fondazione G. Pascale”, 80131 Naples, Italy; m.damico@istitutotumori.na.it (M.D.); 9 Ente Nazionale di Previdenza e Assistenza a Favore dei Biologi (ENPAB), 00153 Rome, Italy; tiziana.stallone@gmail.com (T.S.)

**Keywords:** body composition, machine learning, sarcopenia risk, health-related quality of life, cancer prognosis

## Abstract

**Background/Objectives**: Cancer and related treatments can impair body composition (BC), increasing the risk of malnutrition and sarcopenia, poor prognosis, and Health-Related Quality of Life (HRQoL). To enhance BC parameter interpretation through Bioelectrical Impedance Analysis (BIA), we developed a predictive model based on unsupervised approaches including Principal Component Analysis (PCA) and k-means clustering for sarcopenia risk in cancer patients at the Istituto Nazionale Tumori IRCCS “Fondazione G. Pascale” (Naples). **Methods**: Sarcopenia and malnutrition risks were assessed using the NRS-2002 and SARC-F questionnaires, anthropometric measurements, and BIA. HRQoL was evaluated with the EORTC QLQ-C30 questionnaire. PCA and clustering analysis were performed to identify different BC profiles. **Results:** Data from 879 cancer patients (mean age: 63 ± 12.5 years) were collected: 117 patients (13%) and 128 (15%) were at risk of malnutrition and sarcopenia, respectively. PCA analysis identified three main components, and k-means determined three clusters, namely HMP (High Muscle Profile), MMP (Moderate Muscle Profile), and LMP (Low Muscle Profile). Patients in LMP were older, with a higher prevalence of comorbidities, malnutrition, and sarcopenia. In the multivariable analysis, age, lung cancer site, diabetes, and malnutrition risk were significantly associated with an increased risk of sarcopenia; among the clusters, patients in LMP had an increased risk of sarcopenia (+62%, *p* = 0.006). **Conclusions**: The NUTRISCREEN project, part of the ONCOCAMP study (ClinicalTrials.gov ID: NCT06270602), provides a personalized nutritional pathway for early screening of malnutrition and sarcopenia. Using an unsupervised approach, we provide distinct BC profiles and valuable insights into the factors associated with sarcopenia risk. This approach in clinical practice could help define risk categories, ensure the most appropriate nutritional strategies, and improve patient outcomes by providing data-driven care.

## 1. Introduction

Cancer remains one of the greatest global health challenges, significantly impacting millions of lives each year. In 2022, an estimated 20 million new cancer cases were diagnosed, leading to 9.7 million deaths. Additionally, the number of individuals who survived at least five years after a cancer diagnosis reached 53.5 million. With approximately one in five people developing cancer during their lifetime, and the disease accounting for the death of roughly one in nine men and one in twelve women, the urgency of understanding and addressing its long-term effects becomes evident [1,2]. Beyond its direct impact on survival, cancer and its treatments profoundly affect body composition (BC) and muscle strength (MS), which, in turn, significantly influence patients’ Quality of Life (QoL). Loss of muscle mass (MM), reduced strength, and metabolic alterations can impair physical function, increase fatigue, and limit daily activities.

Cancer patients could experience significant changes in dietary intake and nutritional status, increasing their vulnerability to malnutrition and functional decline. Malnutrition and sarcopenia are major concerns for cancer patients, impacting both survival and treatment outcomes. Factors such as cancer type, disease stage, systemic inflammation, and treatment side effects further heighten the risk of malnutrition and sarcopenia [3]. Malnutrition, driven by cancer-induced inflammation, leads to appetite loss, tissue breakdown, and significant weight reduction, all of which compromise BC and physical performance [4]. Sarcopenia, the progressive loss of MM and physical function, weakens strength, reduces mobility, and increases fatigue, making daily activities more difficult. As sarcopenia progresses, patients may lose their independence and experience a sharp decline in QoL. It is also associated with higher complication risks, poorer treatment responses, and a worse overall prognosis [5]. Consequently, early identification of malnutrition or sarcopenia risk is a crucial part of the oncological patient’s care. 

Patients with advanced cancer are particularly susceptible to cancer-related nutritional disturbances, including malnutrition, reduced MM, sarcopenia, and cachexia, which is defined by ongoing weight loss and progressive reduction in skeletal muscle mass (SMM), with or without fat mass (FM) loss [6]. However, growing evidence suggests that these complications are not limited to metastatic disease. Early-stage, non-metastatic cancer patients may also experience significant nutritional impairments, such as weight loss and muscle depletion, which could influence long-term clinical outcomes [7].

BC assessment plays a crucial role in evaluating the overall nutritional status of cancer patients, as it may directly affect the efficacy and toxicity of anticancer therapies [8,9]. Moreover, impairment in BC has been associated with poorer clinical outcomes, including functional status, surgical complication rates, length of hospital stay, and overall survival. BC can be assessed using various methods, with Bioelectrical Impedance Analysis (BIA) emerging as a practical and widely accessible technique [10]. BIA estimates body fat in relation to lean body mass by measuring the impedance of a low-level electrical current through the body. Compared to imaging methods, BIA is more cost-effective and easier to implement, making it a valuable tool for BC assessment in the clinical setting [11,12]. Other methods to estimate BC include Dual-Energy X-Ray Absorptiometry (DXA), Computed Tomography (CT), and Magnetic Resonance Imaging (MRI). While CT and MRI are gold standards for MM assessment, they are costly and may involve radiation. DXA is more affordable but has limitations [13]. These methods remain widely used in oncology for tumor staging and monitoring. Techniques such as BIA, widely used in clinical nutritional research, enable the assessment of a vast amount of data, including Phase Angle (PhA), FM, and Total Body Water (TBW). These variables provide a comprehensive evaluation of BC, allowing for the correlation of extensive BIA-derived parameters with clinical outcomes in cancer patients. PhA is derived from the relationship between the resistance (R) and reactance (Xc) of body tissues during BIA. It plays a crucial role in assessing nutritional and overall health [14]. PhA reflects cellular integrity, Body Cell Mass (BCM), and the balance between Extracellular Water and Intracellular Water (ECW and ICW). PhA is influenced by factors such as age, gender, and physical activity, with higher values indicating better cellular function and MS. Notably, PhA is a significant prognostic factor, with lower values associated with poorer outcomes in chronic diseases, including cancer, frailty, disability, and mortality [15,16,17]. PhA, as recommended by various guidelines, has been the focus of numerous studies examining its connection to MM and function, as well as sarcopenia. These studies highlight the PhA’s potential as a valuable diagnostic tool for sarcopenia [18,19,20]. Due to its non-invasive nature and ease of measurement through BIA, PhA is increasingly recognized as a useful indicator for identifying individuals at risk of sarcopenia and related health issues.

Data in the field of nutrition are becoming increasingly complex and high-dimensional, driven by the integration of diverse sources such as nutritional screening information, dietary intake, microbiome analysis, and clinical parameters. These datasets often involve numerous variables and intricate relationships, making traditional analytical methods ineffective in studying meaningful insights. As a result, further techniques are essential to extract information from such data to understand the leading behavior of the overall phenomenon. Machine learning (ML) methods, along with unsupervised approaches like Principal Component Analysis (PCA) and k-means clustering, allow researchers to manage this complexity by reducing dimensionality, identifying patterns, and grouping similar data points. These tools enable more accurate predictions and personalized approaches in both research and clinical practice [21]. By utilizing predictive analytics, ML algorithms can model the likelihood of disease development, complications, or hospital admissions/readmissions, allowing healthcare providers to early identify patients at risk. This proactive approach enables tailored, timely interventions that improve patient care, to optimize healthcare resource allocation, and ensure effective management of both medical and nutritional aspects of health. As ML and artificial intelligence (AI) become increasingly integrated into clinical workflows, understanding their principles and applications will be crucial to realizing their full potential in improving clinical decision-making and patients’outcomes [22].

The goal of this study is to apply an unsupervised approach to analyze the growing complexity and volume of data from nutritional and clinical assessments of cancer patients participating in the NUTritional and sarcopenia RIsk SCREENing project (NUTRISCREEN) launched by the INT IRCCS “Fondazione G. Pascale” of Naples with the aim of more accurately identifying risk factors related to malnutrition and sarcopenia. Utilizing methods such as PCA and k-means clustering, this study seeks to uncover underlying patterns and correlations across multiple parameters, thereby improving the personalization of nutritional strategies and therapeutic interventions. Ultimately, this approach aims to enhance patient outcomes by providing more precise, data-driven care.

## 2. Materials and Methods

### 2.1. Study Design

**Figure 1:**
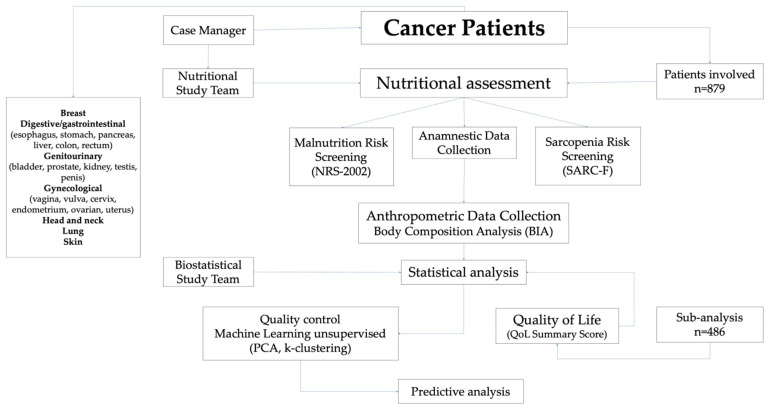
Schematic representation of the study workflow.

The NUTRISCREEN project is part of the ONCOCAMP research protocol, a retrospective–prospective observational, multicenter study (ClinicalTrials.gov ID: NCT06270602 on 21 February 2024) aimed to enhance the appropriateness, effectiveness, and safety of diagnostic and therapeutic pathways, clinical strategies, and procedures within the Campania Oncology Network (Rete Oncologica Campana, ROC) [23]. In this context, the NUTRISCREEN project was launched by the INT IRCCS “Fondazione G. Pascale” of Naples, with the aim of assessing the nutritional status of cancer patients at diagnosis, during the intake and management phase by the Multidisciplinary Oncology Groups (MOGs). This initiative involved the collaboration of Ente Nazionale di Previdenza e Assistenza a Favore dei Biologi (ENPAB, Rome, Italy) and multiple departments, including clinical nutrition biologists, physicians, the Dietetics and Artificial Nutrition Unit, the Epidemiology and Biostatistics Unit, as well as MOG coordinators and case managers. The primary objective of the NUTRISCREEN project is to identify the risks of malnutrition and sarcopenia in cancer patients using scientifically validated assessment tools. By detecting these risks early in the treatment process, the project aims to improve patient outcomes by implementing targeted interventions. These include personalized lifestyle modifications and the introduction of tailored, evidence-based nutritional strategies, ultimately enhancing the patient’s QoL and prognosis throughout their cancer care journey (Figure 1). This study was conducted in accordance with the Declaration of Helsinki, and approved by the Ethics Committee of the Istituto Nazionale Tumori IRCCS “Fondazione G. Pascale” of Naples (registration number 6/23 OSS “ONCOCAMP” on 10 March 2023).

### 2.2. Data Collection

Data from patients enrolled between July 2023 and July 2024 were collected during the first nutritional visit at our cancer center, using a personalized Case Report Form designed by study staff. For this analysis, patients who had not received prior nutritional support or therapy, with a first diagnosis of cancer as well as metastasis and disease recurrence, were included, while patients with missing data were excluded. The following variables were used: gender (male or female), age (years), cancer type (head and neck, breast, digestive/gastrointestinal, genitourinary, gynecological, lung, and skin), education level (1st grade or less, middle school, high school, and graduation), smoking status (non-smoker, former smoker, or smoker), presence of intermediate risk factors or comorbidities (hypertension, diabetes, cardiovascular disease, dyslipidemia, hypercholesterolemia, and other comorbidities) and surgical status (yes or no). Digestive/gastrointestinal cancers included esophagus, stomach, pancreas, liver, colon, and rectum sites; genitourinary included bladder, prostate, kidney, testis, and penis sites; and gynecological included vagina, vulva, cervix, endometrium, ovarian, uterus sites.

### 2.3. Screening Tools: Malnutrition and Sarcopenia Risk

To assess malnutrition risk, we used a validated questionnaire: the Nutritional Risk Screening 2002 (NRS-2002). This tool is designed to identify patients at risk of malnutrition in hospital settings by evaluating two components: nutritional status assessed through Body Mass Index (BMI), weight loss, dietary intake reduction, and disease severity (reflecting the impact of acute or chronic illness on nutritional requirements). The NRS-2002 scoring system ranges from 0 to 7, with a score of ≥3 indicating a higher risk of malnutrition and the need for tailored nutritional intervention [24]. In addition, to evaluate the risk of sarcopenia, we used a simple and effective screening tool, the Strength, Assistance with walking, Rising from a chair, Climbing stairs, and Falls (SARC-F) questionnaire which represents the five key areas evaluated. SARC-F consists of five questions assessing muscle strength, physical performance, and functional ability, each with three possible responses: “No difficulty”, “Some difficulty”, or “Severe difficulty or inability to perform the task”. For each of these responses, a score of 0, 1, or 2, was assigned, respectively. The total score ranges from 0 to 10, with higher scores indicating a greater risk of sarcopenia. A score of 4 or higher is typically considered indicative of sarcopenia risk, warranting further diagnostic evaluation and potential intervention [25]. In our sample, a satisfactory reliability was found (standardized Cronbach’s alpha = 0.79).

### 2.4. Anthropometric Measurements and BC Analysis

Anthropometric measurements, including BMI, waist circumference (WC), and hip circumference (HC), were collected. BC was assessed using BIA with an Akern 101 bioimpedance device. (BIA 101 BIVA® PRO, Akern Srl, Pontassieve, Italy) The BIA measurements were taken at a frequency of 50 Hz, with electrodes placed at four points on the body (hands and feet). The measurements were performed while the patient was in a supine position, in a fasting state, and in the morning to ensure consistency and accuracy of results. This method allowed estimating key components of BC, FM, fat-free mass (FFM), BCM, SMM, appendicular skeletal muscle mass (ASMM), and the ECW/ICW. Subsequently, relative indices for muscle and FM were calculated, accounting for the subject’s height, to assess the distribution and proportion of these components. Additionally, PhA, a marker of cell membrane integrity and nutritional status, was derived from the BIA measurements, as the arctangent of the ratio between Xc and R. The units of measurement for anthropometric parameters included BMI (in kg/m^2^), WC, and HC in centimeters (cm). BMI was calculated using the formula weight (kg)/height (m^2^), according to the WHO recommendations [26]. For BIA indices, FM, FFM, BCM, SMM, ASMM, and ECW/ICW were measured in kilograms (kg), except for ECW/ICW. PhA was measured in degrees (°), and R and Xc in ohms (Ω). For all BIA-derived indices, such as Fat Mass Index (FMI), Fat-free Mass Index (FFMI), Body Cell Mass Index (BCMI), Skeletal Muscle Index (SMI), and Appendicular Skeletal Muscle Index (ASMI), the measurements were adjusted for height squared (m^2^).

### 2.5. Evaluation of HRQoL

HRQoL was evaluated through the European Organization for Research and Treatment of Cancer Quality of Life Questionnaire C30 (EORTC QLQ-C30), which is a validated, cancer-specific questionnaire. The C30 Summary Score (SumSc), ranging from 0 to 100, acts as a comprehensive index reflecting a patient’s overall QoL. It is calculated as the average of the functional and symptom scales from the EORTC QLQ-C30 questionnaire, following an established methodology [27,28]. To maintain consistency across scales, symptom scores were adjusted by subtracting them from 100, ensuring that higher values uniformly indicate better health status. The SumSc was determined by adding the non-missing scores from the five functional scales (physical, role, cognitive, social, and emotional functioning) and the adjusted scores from the seven symptom scales (fatigue, pain, nausea and vomiting, dyspnea, insomnia, appetite loss, constipation, and diarrhea). The final score was obtained by dividing this sum by the number of non-missing values (up to 13) while excluding scores related to global health status/QoL and financial difficulty [29,30].

### 2.6. Statistical Analysis

Initially, a preliminary analysis was conducted to identify potential outliers among BIA variables. Cancer patients with measurements falling below the 0.5th percentile or above the 99.5th percentile were excluded from the subsequent analysis. An explorative correlation analysis among BIA variables was assessed to measure the strength of their interrelationships. A PCA [31,32] and a clustering analysis via k-means algorithm were performed to classify patients according to BC parameters and identify different BC profiles. PCA is a statistical method that transforms a set of correlated continuous variables into a smaller, uncorrelated subspace, preserving as much of the original data’s variability as possible. BIA measures were automatically unit-scaled when fed to the PCA function. K-means, in turn, is an unsupervised ML technique that aims to automatically partition a set of n points in Euclidean space, ensuring that observations within each group are similar, while those in different groups are distinct. The number of groups to reach is given a priori, even if multiple runs still ensure the best number to feed the algorithm as a definitive set. The main Factor Loadings (FLs) were output to detect the main relations between Principal Components (PCs) and the variables.

Univariable analysis and multivariable logistic analysis were conducted to investigate the associations between sarcopenia risk, patient characteristics, and BC profiles. The univariable analysis focused on examining crude associations between SARC-F and key clinical measures, as well as performing correlation analysis among BIA variables to assess the strength of their interrelationships. Pairwise comparisons were adjusted using the Benjamini–Hochberg procedure to account for multiple testing errors. The logistic model was constructed using a stepwise selection approach (stepwise model). The generalized variance inflation factors (GVIFs) were presented to assess the possibility of multicollinearity across the covariates within the model [33]. Although the prevalence of SARC-F ≥ 4 was 15% in our sample, a balanced data partition was ensured. A subgroup analysis was performed on participants who had fully completed the C30 questionnaire. For this purpose, two subgroups were created using the median split of SumSc. The distribution of the sub-sample based on this split, as well as the main adjusted associations between SARC-F and the covariates identified in the stepwise model, were analyzed using stratified logistic models. The SumSc was calculated using the following formula: (13 − k)^−1^ Σ (Physical Functioning, Role Functioning, Social Functioning, Emotional Functioning, Cognitive Functioning, (100 − Fatigue), (100 − Pain), (100 − Nausea and Vomiting), (100 − Dyspnea), (100 − Insomnia), (100 − Appetite Loss), (100 − Constipation), and (100 − Diarrhea)), where *k* represents the number of missing responses. The McFadden R-squared value [28], the area under the curve (AUC), the accuracy value, and the F1 score were reported as global performance metrics for the sample set. The statistical univariate tests included Pearson’s Chi-squared test for contingency tables, the non-parametric Wilcoxon rank sum test for mean differences, and Pearson’s correlation test for linear associations. Bartlett’s test of sphericity [34] and the Kaiser–Meyer–Olkin (KMO) criterion [35] were used to assess the suitability of the data for PCA.

In the subsequent clustering step, the Hopkins statistic and test [36] were applied to evaluate the clustering tendency of the dataset while the Dunn index and the average silhouette score were adopted to show the cluster quality: higher statistics generally indicate a good clusterization of data. The Benjamini–Hochberg procedure (i.e., the False Discovery Rate (FDR)) [37] was applied for multiple hypothesis testing to identify between-group and within groups clusters based on the median split of SumSc among the set of variables while controlling the overall Type I error rate. Logistic model estimates were adjusted using the quasi-variance method for floating absolute risks [38]. All tests and estimates were computed at a 95% significance level. Statistical analyses, graphs, and diagrams were performed using R software, version 4.4.0 (R Core Team, 2024), along with the following packages and versions: car (3.1-3), fpc (2.2-13), hopkins (1.1), EFAtools (0.4.4), FactoMineR (2.11), factoextra (1.0.7), ggpubr (0.6.0), ggplot2 (3.5.1), cowplot (1.1.3), janitor (2.2.1), tidyr (1.3.1), dplyr (1.1.4), readxl (1.4.3), stringr (1.5.1), lubridate (1.9.3), gt (0.11.0), flextable (0.9.6), gtsummary (2.0.2), psych (2.4.6.), epi (2.59). Graphs were exported in high resolution (minimum 300 dpi) to ensure clarity and print quality, in accordance with the publisher’s guidelines.

## 3. Results

### 3.1. Descriptive Results

Data from 879 cancer patients were collected. Fifty-six percent were males, the mean age was 63 years (±12.5), 54% had a high education level (high school or higher degree), and 28% were smokers. The most prevalent cancer types were digestive/gastrointestinal (35%) and genitourinary (22%). Almost half (45%) had hypertension, 16% had diabetes, and 9% and 5% of patients suffered from cardiovascular disease and dyslipidemia, respectively. Finally, 21% of patients had hypercholesterolemia and 4% reported additional comorbidities. In the overall sample, 117 patients (13%) were at high nutritional risk according to NRS-2002, while 128 (15%) were at high risk of sarcopenia assessed by SARC-F. Descriptive statistics, including percentages and counts, are provided in Table 1a.

### 3.2. Univariable Analysis

The results of univariable analysis, reported in Table 1a, indicated significant associations between sarcopenia risk, age, and cancer type. Patients with SARC-F ≥ 4 were older (70 vs. 62, *p* < 0.001) and had a higher prevalence of digestive/gastrointestinal and lung cancers (44.5% and 34.4%) compared to patients with SARC-F < 4 (33.8% and 9.7%, respectively, with a *p*-value from the Pearson’s Chi-squared test < 0.001). Other significant associations were found for socio-demographic factors and comorbidities.

Table 1b shows descriptive results and univariate analysis regarding patients’ nutritional status according to sarcopenia risk. The subsample of patients at high risk of malnutrition (NRS-2002 ≥ 3) in the group at risk of sarcopenia was 37.5% vs. 9.2% in the subgroup of patients with SARC-F < 4 (*p* < 0.001). BIA-derived indices including PhA, BCMI, ASMI, and ECW/ICW were individually associated with SARC-F.

### 3.3. Evaluation of BMI Across Age, Sex, and Cancer Types

An initial classification of patients’ nutritional status was performed using BMI, as it is widely recognized as a simple measure to categorize patients’ weight status in the function of height (underweight, normal weight, overweight, or obesity). While useful for identifying potential health risks at the population level, BMI does not capture differences in MM and FM. Therefore, its calculation was complemented with more specific measures, like BIA, for a more detailed assessment. BMI was assessed across several variables, including age groups (≤65 years and >65 years), sex (female and male), and cancer type (head and neck, breast, digestive/gastrointestinal, genitourinary, gynecological, lung, and skin). No significant differences were found between age groups. Mean BMI was 27.43 (SD = 5.51) for patients aged 65 years or younger and 27.06 (SD = 4.88) for patients older than 65 years. The proportions of participants in the different BMI categories—normal weight, overweight, and obesity—were also similar across the two age groups (Table S1). The distribution of BMI categories between genders was comparable, with no significant differences observed (Table S2).

However, when examining BMI across different cancer types, significant differences were observed (Table S3). Mean BMI ranged from 26.07 (SD = 4.32) for digestive/gastrointestinal cancer to 29.13 (SD = 6.94) for gynecological cancer (*p* < 0.001). Moreover, the distribution of BMI categories varied notably according to cancer type: genitourinary cancer had the highest percentage of individuals with BMI ≥ 30 (32.86%), while digestive/gastrointestinal cancer had the highest percentage of individuals with BMI < 25 (44.80%) (*p* < 0.001).

### 3.4. Correlation Analysis Between BIA-Derived Parameters

**Figure 2:**
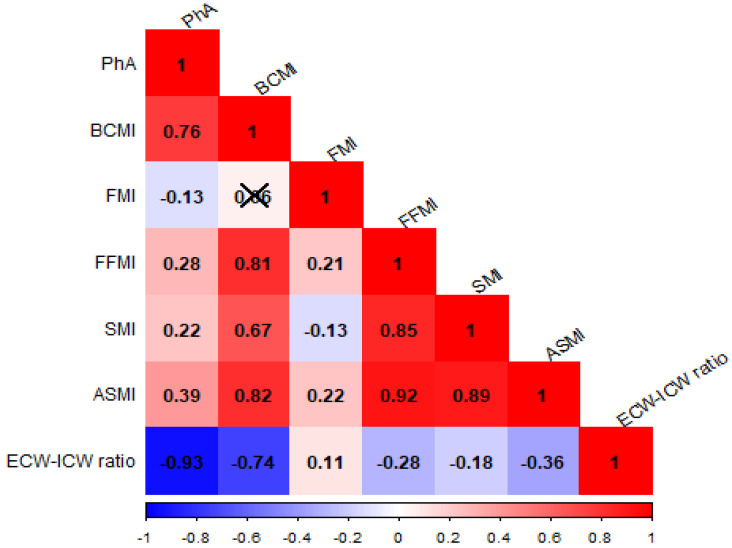
Correlation analysis among the BIA parameters. The horizontal axis indicates the direction and intensity of the correlation coefficient between the two variables intersecting at the single cell. Cross refers to not significant Person’s correlation coefficients (ρs). PhA, Phase Angle; BCMI, Body Cell Mass Index; FMI, Fat Mass Index; FFMI, Fat-free Mass Index; SMI, Skeletal Muscle Index; ASMI, appendicular skeletal muscle mass; and ECW:ICW ratio, Extracellular Water–Intracellular Water ratio.

Figure 2 shows the correlation pattern between BIA-derived parameters: strongest relations (ρ > 0.5) were found between PhA with ECW/ICW (ρ = −0.93) and BCMI (ρ = 0.76), between ASMI with FFMI (ρ = 0.92), SMI (ρ = 0.92), and BCMI (ρ = 0.82), between SMI and FFMI (ρ = 0.85), and between SMI and BCMI (ρ = 0.67). These correlations were statistically significant (*p*-value from Pearson’s correlation test < 0.01, for all the comparisons). Data from the BIA measures were suitable for factor analysis (KMO statistic = 0.60, *p* < 0.01; and *p* < 0.01 from Bartlett’s test of sphericity). Figure S1 shows the density shapes of BIA parameters and the associated results from normality tests.

### 3.5. PCA and k-Means: Unsupervised ML Approaches

**Figure 3:**
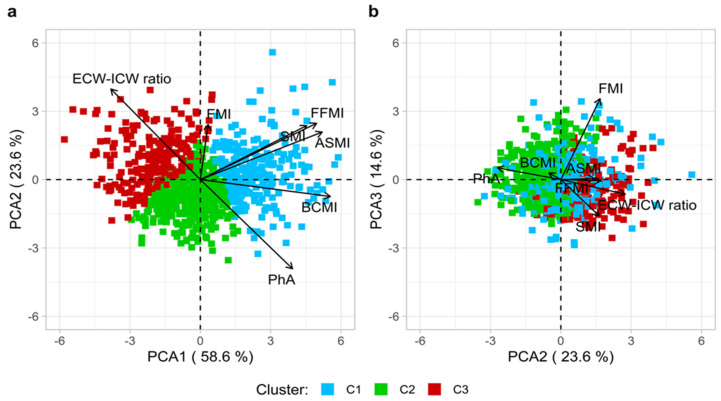
PCA results and k-means clustering outputs for the most important dimensions ((**a**) 1st and 2nd dimensions; (**b**) 2nd and 3rd dimensions). C1 = HMP (High Muscle Profile), C2 = MMP (Moderate Muscle Profile), and C3 = LMP (Low Muscle Profile). The overall explained variance was almost 97%. PhA, Phase Angle; BCMI, Body Cell Mass Index; ASMI, Appendicular Skeletal Muscle Index; SMI, Skeletal Muscle Index; FFMI, Fat-free Mass Index; FMI, Fat Mass Index; and ECW-ICW ratio, Extracellular Water–Intracellular Water ratio.

PCA analysis identified three main components, with explained variance distributed as follows: PC1 (59%), PC2 (24%), and PC3 (15%) (Figure 3). The first dimension was primarily driven by most parameters (FLs: BCMI = 0.48, ASMI = 0.45, FFMI = 0.43, and SMI = 0.39). The second dimension was associated with the ECW/ICW ratio and PhA (Factor Loadings: 0.54 and −0.53, respectively). Lastly, PC3 was mainly explained by FMI (FL = 0.89) and, to a lesser extent, by SMI (FL = −0.40). The Hopkins statistic of 0.77 (bootstrap *p* < 0.01) indicated a well-defined clustering structure in the data, supporting the application of clustering techniques. The k-means clustering analysis partitioned the data into three distinct clusters (C1: *n* = 253, C2: *n* = 410, and C3: *n* = 216; see the elbow plot in Figure S2). Clusters C1, C2, and C3 were labeled based on key differences in BC parameters, including MM, cellular integrity, and fluid distribution. These distinctions led to the identification of three profiles: High Muscle Profile (HMP) for C1, Moderate Muscle Profile (MMP) for C2, and Low Muscle Profile (LMP) for C3, reflecting progressively lower muscle and cellular quality across the cluster (Figure 3). Notably, the Dunn index was found to be very low (=0.02) but formally among the highest values across the explored partitions. Finally, the average silhouette score from the three clusters was 0.26, which tendentially referred to the medium goodness of the obtained clusterization (Figure S2).

According to Table 2a, patients in HMP were predominantly male (87% vs. 40.5% in MMP and 48.6% in LMP, *p* < 0.001), whereas patients in LMP were older (69 years vs. 62 years in MMP and 61 years in HMP *p* < 0.001). Hypertension was more frequent in LMP (53.2% vs. 40.5% in MMP and 44.3% in HMP, *p* = 0.015); LMP also had a higher prevalence of patients with one or more comorbidities (30% vs. an overall average of 20%, *p* = 0.015).

Regarding nutritional status, patients in LMP were more frequently at high risk of malnutrition (NRS-2002 ≥ 3: 22.7% vs. 10.7% in MMP and 9.5% in HMP, *p* < 0.001) and sarcopenia (24.5% vs. 12% in MMP and 10.3% in HMP, *p* < 0.001), and were characterized by worse BIA-related parameters. Notably, HMP exhibited the best overall performance in BIA measures (*p* < 0.001 for all, except FMI) (Table 2b).

### 3.6. Multivariable Analysis

Since patients with skin cancer (*n* = 47) were at low risk of sarcopenia (SARC-F < 4), they were excluded from the analysis, leaving a final sample of 832 patients. The stepwise model was not statistically different from the maximal model (*p* = 0.76 from the Wald test), with a similar McFadden R-squared value of 0.26.

The covariates included in the analysis were age (an increase of 5 years, *p* from the z-test < 0.01), cancer type, education, presence of diabetes or other comorbidities, malnutrition risk (NRS-2002 ≥ 3), and BC profiles. For these measures, the *p*-value from the Wald test was <0.01. Other features included undergoing surgery (*p* = 0.01) and dyslipidemia (*p* = 0.04). The terms included in the model were not strongly related to the others (GVIF < 1.9 for all covariates), indicating an almost absent multicollinearity issue within the accounted model. Trends were significant for both education and cluster (for all, *p* < 0.01). All estimates can be found in Table 3. For each 5-year increase in age, sarcopenia risk increased by 17% (95% CI: 1.10, 1.25). Regarding cancer type, digestive/gastrointestinal, gynecological, and lung cancers were positively associated with sarcopenia risk. Specifically, lung cancer had an eleven-fold increased risk (95% CI: 8.31, 14.5) of sarcopenia compared to head and neck cancer, followed by gynecological cancer (OR = 5.0, 95% CI: 3.16, 7.82) and digestive/gastrointestinal cancer (OR = 2.71, 95% CI: 2.12, 3.46). Educational level was identified as a protective factor for sarcopenia risk across all categories, with a particularly strong association for patients with middle school education (OR = 0.53, 95% CI: 0.42, 0.66), high school (OR = 0.38, 95% CI: 0.31, 0.48), and higher education levels (OR = 0.32, 95% CI: 0.22, 0.47, *p* < 0.001). Patients with diabetes had an increased risk of sarcopenia (OR = 1.70, 95% CI: 1.23, 2.36, *p* = 0.001). Moreover, patients at high malnutrition risk had a 5-fold increased risk of sarcopenia (95% CI: 3.38, 6.93, *p* < 0.001). Finally, LMP had a higher sarcopenia risk (OR = 1.62, 95% CI: 1.26, 2.07) compared to HMP.

### 3.7. A Subgroup Analysis: SumSc and Clinical Associations in Cancer Patients

This subgroup analysis included a total of 486 patients with complete data on C30. Median SumSc was 84 (IQR = 69.9, 92.0) with a minimum value of 18. Twenty cancer patients reported a score of 100, which is the maximum value, resulting in a strongly right-skewed asymmetric distribution.

Univariate analysis was performed by stratifying patients into two groups based on the median value of the SumSc (≤84 and >84) (Table S4). These results indicated that men tended to have a higher SumSc than women (*p* = 0.014). Significant differences were observed across cancer types (*p* = 0.002), education levels (*p* = 0.008), the presence of diabetes (*p* = 0.011), cardiovascular disease (*p* = 0.008), or other comorbidities (*p* < 0.001). Similarly, patients at risk of malnutrition or sarcopenia reported less frequently SumSc values > 84 (3.4% vs. 21.7% for malnutrition risk, 5.2% vs. 24.7% for sarcopenia risk, *p* < 0.001 for both). In terms of classification, HMP was predominantly represented by patients with SumSc > 84 (30.5 vs. 21.3), whereas LMP exhibited the opposite trend (*p* = 0.014).

The multivariable, stratified analysis was performed on patients at risk of sarcopenia (SARC-F ≥ 4) according to median SumSc (≤84 and >84) (Table S5). Among patients with SumSc ≤ 84 (*n* = 235), cancer type and education level retained the same directional trends observed in the overall analysis (*p* < 0.001 and *p* = 0.005, respectively), although the neoplasm coefficients were likely inflated due to the limited sample size. Moreover, patients at risk of malnutrition and with SumS ≤ 84 also had an increased risk of sarcopenia (OR = 2.34, 95% CI: 1.30, 4.32). Moreover, in this subgroup of patients with SumSc ≤ 84, associations with diabetes (which was associated with sarcopenia risk in the univariable analysis, *p*=0.011), dyslipidemia, and number of comorbidities were not statistically significant. Overall, cluster classification was not significantly associated with sarcopenia (*p* = 0.354); however, individually, patients with LMP and SumSc ≤ 84 had an increased risk of sarcopenia (OR = 1.62, 95% CI: 1.05, 2.50, *p*-value from the Wald test = 0.35).

Since at univariate analysis, it was observed that patients with diabetes, dyslipidemia, or multiple comorbidities reported less frequently a SumSc > 84, the first two variables were excluded from the logistic model involving patients with SumSc > 84, and participants with more comorbidities were grouped together.

In this analysis, we found that increased age (evaluated as a 5-year increase) was associated with a higher risk of sarcopenia (*p* = 0.015). Patients with comorbidities and SumSc > 84 had an increased risk of sarcopenia (OR = 3.61, 95% CI: 1.73, 7.56). Similarly, patients at high risk of malnutrition and SumSc > 84 had a five-fold increased risk of sarcopenia (*p* < 0.009). Finally, patients with LMP and SumSc > 84 had a 2.5 times higher risk of sarcopenia compared to patients with HMP (95% CI: 1.43, 4.47, *p* < 0.008).

## 4. Discussion

Our analysis indicates that the risk of sarcopenia in cancer patients is significantly influenced by a combination of demographic, clinical, and nutritional factors. Advanced age, specific cancer types (such as lung, gynecological, and gastrointestinal cancers), and the presence of comorbidities, particularly diabetes and malnutrition, are strongly associated with an increased risk of sarcopenia. Conversely, a higher level of education appears to have a protective effect. These results emphasize the need for targeted interventions to monitor and manage sarcopenia risk in vulnerable cancer patient populations.

Sarcopenia is well documented in the literature as a condition strongly correlated with aging, affecting both healthy individuals and clinical populations. Studies show that among healthy individuals aged ≥ 60 years, 32.4% exhibit a low SMI, with prevalence increasing significantly in older age groups [39]. In an Italian cohort, sarcopenia affected 35.3% of males and 10.3% of females aged 66–84 years, rising to 53.7% and 18%, respectively, in those over 84 years [40]. A global meta-analysis further supports this trend, estimating an overall prevalence of 27% in individuals > 60 years [41]. In a recent observational study, age emerged as the most critical risk factor for sarcopenia in both men and women, highlighting its strong impact on muscle deterioration. While other factors such as anemia, BMI, Karnofsky Performance Status, hypoalbuminemia, and reduced food intake also played a role, age remained the predominant determinant [20].

Our findings are consistent with these data, confirming that age is a critical determinant of sarcopenia risk, also in cancer patients. Patients at high risk of sarcopenia were, on average, 8 years older compared to patients not at risk, emphasizing the role of aging in muscle deterioration. Additionally, we observed that the probability of developing sarcopenia increased by 17% for every 5-year increase in age. These results reinforce the established link between sarcopenia and aging, underscoring the importance of early identification and targeted interventions in older individuals, especially patients with cancer, to mitigate sarcopenia-related complications [42].

Both our findings and the previous literature suggest a higher vulnerability to sarcopenia in women. The cited study reports a greater prevalence of sarcopenia in women than in men (26.4% vs. 19.2%) [43], in line with our results indicating that men were more represented in HMP. This reinforces the idea that gender may have a protective effect against sarcopenia, potentially due to hormonal, metabolic, or lifestyle-related factors [43,44]. These aspects warrant further investigation to better understand gender-specific differences in muscle deterioration and sarcopenia risk. A study conducted in 2015 highlights sex-specific differences in the mechanisms underlying sarcopenia. It suggests that in men, sarcopenia is more influenced by myostatin and serum triglycerides, whereas in women, it is driven by anabolic decline, particularly reduced IGF-1 levels, and nutrition [45]. This study provides a deeper biological explanation, emphasizing the need for gender-specific interventions in managing sarcopenia.

In this analysis, the risk of sarcopenia varied according to cancer site: it was higher in patients with lung cancer, followed by gynecological and digestive/gastrointestinal cancer. These results supported previous studies suggesting that sarcopenia is a highly prevalent condition in lung cancer patients, ranging from 42.8% to 45% [46]. Patients with chronic pulmonary disease can experience reduced MS and/or function, involuntary weight loss, and nutritional deficiencies. The detrimental effects on nutritional status tend to coexist and predict worse outcomes, including poor pulmonary function, impaired physical functioning, poor HRQoL, increased healthcare costs, and higher mortality rates [47].

Sarcopenia is also a highly prevalent condition in gynecological cancer patients, ranging from 9.5% to 62.7% [48]. In line with these findings, in our study, patients with gynecological cancer had a five-fold increased risk of sarcopenia. Evidence indicated that older age, BMI greater than 25 kg/m^2^, as well as ovarian and endometrial cancer sites, could represent plausible risk factors for sarcopenia in this patient population [48].

Patients with digestive/gastrointestinal cancer had a three-fold increased risk of sarcopenia compared to other cancer sites. These patients typically experience dysphagia, weight loss, loss of appetite, nausea, vomiting, hematemesis, and impaired liver function. These factors facilitate nutritional insufficiency, which can lead to the development of sarcopenia. Furthermore, treatment of digestive/gastrointestinal cancers involves major resections of the organ, chemotherapy, and radiotherapy, all of which could impair BC [49].

Another important concern is the association between diabetes and sarcopenia risk. Cancer patients are generally exposed to several cancer-specific and non-cancer-specific factors that cause a decrease in MM and function. Among the others, comorbidities, tumor-derived factors, cancer therapy, and supportive medication, as well as chronic inflammation could influence the risk of sarcopenia [50]. In particular, Type 2 diabetes (T2D) is characterized by insulin resistance, chronic inflammation, accumulation of advanced glycation products, and increased oxidative stress, which can negatively affect SMM, strength, and function, leading to sarcopenia. Moreover, T2D could accelerate the decline in MM and function, which, in turn, can lead to impaired glucose metabolism, reduced physical activity, and the risk of diabetes [51]. These findings highlight the need for tailored nutritional intervention aimed at reducing the impact of both sarcopenia and metabolic conditions on patients’ BC and clinical outcomes.

In this study, a subgroup analysis according to the median value of SumSc was performed. This enabled the identification of potential socio-demographic, clinical, and nutritional factors that could affect patients’ HRQoL.

In the multivariable analysis, in contrast to our hypothesis, the presence of comorbidities was not significantly associated with poorer HRQoL (SumSc ≤ 84), although significant differences were found in the univariate analysis. Indeed, we did not find statistically significant differences for diabetes, dyslipidemia, and the number of comorbidities. Conversely, Malhotra et al. found that the presence of thyroid disease and diabetes was associated with poorer HRQoL in patients with advanced cancer [52,53], while Vissers et al. found that variance in physical and emotional functioning as well as in pain and fatigue was mainly explained by comorbidities rather than socio-demographic and clinical characteristics [54]. This discrepancy, in part, could be related to other factors not included in our analysis, such as cancer stage and anticancer treatment [55].

This data-driven methodology, utilizing the combined approach of PCA and k-means clustering, enabled the identification of underlying patterns in BIA-related parameters and the delineation of distinct groups based on sarcopenia risk. This integrated technique has been successfully applied in various clinical studies for risk group identification, underscoring its utility in patient stratification and personalization of therapeutic interventions. For instance, Guedon et al. [56] employed factor analysis on mixed data, followed by hierarchical cluster analysis, to identify phenotypic profiles associated with varying susceptibility to Antiphospholipid Syndrome—specifically the incidence of new venous or arterial thrombosis—as well as obstetric adverse events during follow-up, in a multicenter retrospective cohort study. In a manner analogous to our approach, PCA combined with k-means clustering was applied to a set of blood biomarkers to create risk profiles for metabolic syndrome, which were subsequently integrated into survival analysis models, revealing varying mortality risks across distinct patient groups [57]. Moreover, the k-means algorithm has been uniquely employed across BIA-related parameters to assess cardiovascular disease risk, as demonstrated by a recent study [58]. In that study, researchers calculated the mean percent increase in cardiovascular disease risk, which was assessed via three distinct scores, across five subphenotypes of BC. However, to the best of our knowledge, no published studies have utilized a similar ML approach to assess sarcopenia status and BIA measurements. This represents a novel application of PCA and k-means clustering to analyze BC data in the context of sarcopenia, providing a unique contribution to the literature and offering promising potential for future clinical applications.

By using PCA and clustering in our integrated approach, we provided BC profiles associated with sarcopenia risk, according to BIA-related parameters and indices in a sample of patients with different cancer types. Nowadays, PCA is still a very used tool to explore linear patterns from a set of highly correlated measures. Moreover, we choose to keep the k-means algorithm to perform an unsupervised classification: indeed, k-means is widely recognized for its quality of classification and simplicity and it is highly cited in the literature. After the identification of BC profiles from BIA-related measures through the algorithms, we found that these profiles were highly related to the SARC-F and NRS-2002 questionnaires as a measure of malnutrition risk. This method might be validated to definitely provide a prediction of sarcopenia risk. Despite the innovation, our study has some limitations: specifically, the analysis included all cancer patients enrolled in the NUTRISCREEN project, regardless of their tumor characteristics and anticancer treatments, without differentiating between first diagnosis, disease recurrence, and metastasis. Additionally, in this analysis, we did not include data regarding patients’ dietary habits and physical activity levels, as well as serum biomarkers of malnutrition risk, which could enhance the interpretation of BIA parameters and more accurately define BC profiles.

This limits the generalizability of our findings, as these factors may have influenced patients’ BC, and consequently, the risk of malnutrition and sarcopenia, as well as their HRQoL. Several studies have investigated the role of BC on clinical outcomes in cancer patients, focusing on individual parameters and using gold-standard techniques to identify potential predictors of clinical outcomes [59,60,61]. The identification of BC profiles by combining BIA-related parameters with certain anamnestic and clinical variables could have practical applications in clinical settings, guiding patients towards a personalized and targeted nutritional approach. Furthermore, implementing BC profiles in clinical practice could be valuable for studying their association with key clinical outcomes, such as survival, treatment response rates, surgical complications, and treatment-related toxicities. In support of this hypothesis, quality of life (C30 SumSc) which is inversely related to cancer prognosis, was associated with worse BC profiles in our study.

Further clinical and statistical evaluation will be necessary, including BIA-related parameters, other nutritional indices, and a systematic, well-balanced number of patients with different tumor sites.

## 5. Conclusions

Cancer care requires an integrated, comprehensive, and multidisciplinary approach that incorporates early nutritional risk screening and evaluation as the current oncological guidelines suggest. In this analysis, we first provided a brief description of the nutritional pathway—the NUTRISCREEN project—implemented at the National Cancer Institute IRCCS Fondazione “G. Pascale” of Naples for cancer patients. This project focuses on early screening for malnutrition and sarcopenia risks and guides patients through a standardized nutritional status evaluation within a multidisciplinary care model.

The analysis of data collected during these nutritional evaluations offers the opportunity to enhance the interpretation of body composition parameters and indices and to understand their complex relationship with patients’ clinical outcomes. Using an unsupervised approach with PCA and k-means clustering, we condensed a large set of variables into a single cluster, identifying three distinct body composition profiles. Analyzing these profiles provides valuable insights into the factors associated with sarcopenia risk. Additionally, classifying patients by their body composition profile in clinical practice could help define risk categories and ensure the most appropriate nutritional and therapeutic strategies. This approach aims to improve patient outcomes by providing more precise, data-driven care. Early identification of malnutrition and sarcopenia, followed by timely intervention, is critical for improving clinical outcomes and quality of life. Our analysis supports this relationship, as patients with impaired body composition higher malnutrition and sarcopenia risk, had lower quality of life. To confirm these results, further studies with larger cohorts are necessary.

## Figures and Tables

**Table 1:** (**a**) Sample characteristics according to sarcopenia risk (SARC-F ≥ 4). (**b**) Nutritional characteristics according to sarcopenia risk.

(**a**)				
	**Sample Characteristics**	**Univariable Analysis**		
**Variable**	**Overall (*n* = 879)**	**SARC-F < 4, ** ***n* = 751 (85%)**	**SARC-F ≥ 4,** ***n* = 128 (15%)**	*p*-Value *
Gender, *n* (%)				0.890 ^1^
Female	388 (44.1%)	330 (43.9%)	58 (45.3%)	
Male	491 (55.9%)	421 (56.1%)	70 (54.7%)	
Age (ys)				**<0.001 ^2^**
Mean (SD)	63.1 (12.5)	61.9 (12.3)	70.2 (11.0)	
Median (IQR)	64.7 (54.7, 72.9)	63.3 (53.5, 71.7)	72.3 (64.7, 77.6)	
Neoplasm, *n* (%)				**<0.001 ^1^**
Head and neck	43 (4.9%)	40 (5.3%)	3 (2.3%)	
Breast	91 (10.4%)	85 (11.3%)	6 (4.7%)	
Digestive/gastrointestinal	311 (35.4%)	254 (33.8%)	57 (44.5%)	
Genitourinary	195 (22.2%)	186 (24.8%)	9 (7.0%)	
Gynecological	75 (8.5%)	66 (8.8%)	9 (7.0%)	
Lung	117 (13.3%)	73 (9.7%)	44 (34.4%)	
Skin	47 (5.3%)	47 (6.3%)	0 (0.0%)	
Civil status, *n* (%)				**0.002 ^1^**
Bachelor	86 (9.8%)	83 (11.1%)	3 (2.3%)	
Married/cohabiting	663 (75.4%)	565 (75.2%)	98 (76.6%)	
Divorced	57 (6.5%)	49 (6.5%)	8 (6.3%)	
Widow	73 (8.3%)	54 (7.2%)	19 (14.8%)	
Education, *n* (%)				**<0.001 ^1^**
Less than 1st grade	136 (15.5%)	93 (12.4%)	43 (33.6%)	
Middle school	272 (30.9%)	228 (30.4%)	44 (34.4%)	
High school	329 (37.4%)	297 (39.5%)	32 (25.0%)	
Graduated	142 (16.2%)	133 (17.7%)	9 (7.0%)	
Smoking status, *n* (%)				0.180 ^1^
No smoking	276 (31.4%)	243 (32.4%)	33 (25.8%)	
Former smoker	360 (41.0%)	297 (39.5%)	63 (49.2%)	
Smoker	243 (27.6%)	211 (28.1%)	32 (25.0%)	
Hypertension, *n* (%) ^a^	393 (44.7%)	320 (42.6%)	73 (57.0%)	**0.006 ^1^**
Diabetes, *n* (%) ^a^	141 (16.0%)	105 (14.0%)	36 (28.1%)	**<0.001 ^1^**
Cardiovascular disease, *n* (%) ^a^	83 (9.4%)	66 (8.8%)	17 (13.3%)	0.210 **^1^**
Dyslipidemia, *n* (%) ^a^	42 (4.8%)	34 (4.5%)	8 (6.3%)	0.660 ^1^
Hypercholesterolemia, *n* (%) ^a^	187 (21.3%)	157 (20.9%)	30 (23.4%)	0.670 ^1^
Cancer surgery, *n* (%) ^a^	135 (15.4%)	115 (15.3%)	20 (15.6%)	>0.990 ^1^
Other comorbidities, *n* (%) ^a^				**<0.001 ^1^**
One	175 (19.9%)	132 (17.6%)	43 (33.6%)	
More	33 (3.8%)	24 (3.2%)	9 (7.0%)	
(**b**)				
	**Sample Characteristics**	**Univariable Analysis**		
**Variable**	**Overall (*n* = 879)**	**SARC-F < 4, ** ***n* = 751 (85%)**	**SARC-F ≥ 4,** ***n* = 128 (15%)**	*p*-Value *
NRS-2002, *n* (%)				**<0.001 ^1^**
<3	762 (86.7%)	682 (90.8%)	80 (62.5%)	
≥3	117 (13.3%)	69 (9.2%)	48 (37.5%)	
PhA				**<0.001 ^2^**
Mean (SD)	5.6 (1.1)	5.7 (1.1)	5.1 (1.2)	
Median (IQR)	5.5 (4.8, 6.2)	5.6 (5.0, 6.3)	5.0 (4.3, 5.8)	
BCMI				**<0.001 ^2^**
Mean (SD)	10.0 (2.0)	10.1 (1.9)	9.4 (2.1)	
Median (IQR)	9.9 (8.6, 11.1)	9.9 (8.7, 11.2)	9.4 (8.1, 10.5)	
FMI				0.670 ^2^
Mean (SD)	7.7 (3.7)	7.7 (3.6)	8.0 (4.3)	
Median (IQR)	7.3 (5.1, 9.9)	7.2 (5.2, 9.7)	7.4 (4.7, 10.5)	
FFMI				0.470 ^2^
Mean (SD)	19.6 (2.5)	19.6 (2.4)	19.4 (2.5)	
Median (IQR)	19.3 (17.9, 21.1)	19.3 (17.9, 21.2)	19.2 (17.7, 20.7)	
SMI				0.210 ^2^
Mean (SD)	9.2 (1.7)	9.2 (1.8)	8.9 (1.6)	
Median (IQR)	9.0 (7.9, 10.2)	9.1 (7.9, 10.3)	8.9 (7.8, 9.7)	
ASMI				**0.013 ^2^**
Mean (SD)	7.4 (1.2)	7.4 (1.2)	7.2 (1.2)	
Median (IQR)	7.3 (6.6, 8.1)	7.3 (6.6, 8.1)	7.1 (6.5, 7.7)	
ECW-ICW ratio				**<0.001 ^2^**
Mean (SD)	1.0 (0.2)	0.9 (0.2)	1.1 (0.3)	
Median (IQR)	0.9 (0.8, 1.1)	0.9 (0.8, 1.0)	1.0 (0.9, 1.2)	

* *p*-values refer to Benjamini–Hochberg correction for multiple test comparisons; ^1^ Pearson’s Chi-squared test; and ^2^ Wilcoxon rank sum test. ^a^ For these variables, only patients with the respective comorbidities or who underwent surgery are reported. Significant *p*-values are shown in bold. NRS-2002, Nutritional Risk Screening 2002; PhA, Phase Angle; BCMI, Body Cell Mass Index; FMI, Fat Mass Index; FFMI, Fat-free Mass Index; SMI, Skeletal Muscle Index; ASMI, Appendicular Skeletal Muscle Index; and ECW-ICW ratio, Extracellular Water–Intracellular Water ratio.

**Table 2:** (**a**) Sample characteristics according to the three clusters’ distribution. (**b**) Nutritional characteristics according to the three clusters’ distribution.

(**a**)				
**Characteristic**	**HMP, *n* = 253**	**MMP, *n* = 410**	**LMP, *n* = 216**	*p*-Value *
Gender, *n* (%)				**<0.001 ^2^**
Female	33 (13.0%)	244 (59.5%)	111 (51.4%)	
Male	220 (87.0%)	166 (40.5%)	105 (48.6%)	
Age (ys)				**<0.001 ^2^**
Mean (SD)	61.6 (11.7)	61.1 (12.6)	68.8 (11.6)	
Median (IQR)	63.3 (54.6, 69.9)	61.8 (52.2, 71.0)	71.4 (63.4, 76.7)	
Neoplasm, *n* (%)				**<0.001 ^1^**
Head and neck	18 (7.1%)	13 (3.2%)	12 (5.6%)	
Breast	9 (3.6%)	59 (14.4%)	23 (10.6%)	
Digestive/gastrointestinal	99 (39.1%)	142 (34.6%)	70 (32.4%)	
Genitourinary	75 (29.6%)	70 (17.1%)	50 (23.1%)	
Gynecological	10 (4.0%)	50 (12.2%)	15 (6.9%)	
Lung	23 (9.1%)	55 (13.4%)	39 (18.1%)	
Skin	19 (7.5%)	21 (5.1%)	7 (3.2%)	
Civil status, *n* (%)				**<0.001 ^2^**
Bachelor	20 (7.9%)	43 (10.5%)	23 (10.6%)	
Married/cohabiting	205 (81.0%)	308 (75.1%)	150 (69.4%)	
Divorced	18 (7.1%)	29 (7.1%)	10 (4.6%)	
Widow	10 (4.0%)	30 (7.3%)	33 (15.3%)	
Education, *n* (%)				**<0.001 ^1^**
Less than 1st grade	31 (12.3%)	52 (12.7%)	53 (24.5%)	
Middle school	97 (38.3%)	107 (26.1%)	68 (31.5%)	
High school	89 (35.2%)	176 (42.9%)	64 (29.6%)	
Graduated	36 (14.2%)	75 (18.3%)	31 (14.4%)	
Smoking status, *n* (%)				0.337 ^1^
No smoking	72 (28.5%)	143 (34.9%)	61 (28.2%)	
Former smoker	107 (42.3%)	158 (38.5%)	95 (44.0%)	
Smoker	74 (29.2%)	109 (26.6%)	60 (27.8%)	
Hypertension, *n* (%)	112 (44.3%)	166 (40.5%)	115 (53.2%)	0.015 ^1^
Diabetes, *n* (%)	44 (17.4%)	53 (12.9%)	44 (20.4%)	0.059 ^1^
Cardiovascular disease, *n* (%)	28 (11.1%)	29 (7.1%)	26 (12.0%)	0.097 ^1^
Dyslipidemia, *n* (%)	17 (6.7%)	17 (4.1%)	8 (3.7%)	0.245 ^1^
Hypercholesterolemia, *n* (%)	48 (19.0%)	85 (20.7%)	54 (25.0%)	0.277 ^1^
Cancer surgery, *n* (%)	30 (11.9%)	67 (16.3%)	38 (17.6%)	0.199 ^1^
Other comorbidities, *n* (%)				**0.015 ^1^**
None	208 (82.2%)	312 (76.1%)	151 (69.9%)	
One	42 (16.6%)	81 (19.8%)	52 (24.1%)	
More	3 (1.2%)	17 (4.1%)	13 (6.0%)	
(**b**)				
**Characteristic**	**HMP, *n* = 253**	**MMP, *n* = 410**	**LMP, *n* = 216**	*p*-Value *
NRS-2002, *n* (%)				**<0.001 ^1^**
<3	229 (90.5%)	366 (89.3%)	167 (77.3%)	
≥3	24 (9.5%)	44 (10.7%)	49 (22.7%)	
SARC-F, *n* (%)				**<0.001 ^1^**
<4	227 (89.7%)	361 (88.0%)	163 (75.5%)	
≥4	26 (10.3%)	49 (12.0%)	53 (24.5%)	
PhA				**<0.001 ^1^**
Mean (SD)	6.3 (1.0)	5.8 (0.7)	4.2 (0.5)	
Median (IQR)	6.1 (5.6, 6.9)	5.7 (5.3, 6.2)	4.3 (3.9, 4.6)	
BCMI				**<0.001 ^1^**
Mean (SD)	12.2 (1.4)	9.7 (0.9)	7.8 (1.0)	
Median (IQR)	12.0 (11.3, 13.0)	9.8 (9.1, 10.4)	8.0 (7.2, 8.6)	
FMI				0.165 ^1^
Mean (SD)	8.1 (4.1)	7.5 (3.5)	7.8 (3.5)	
Median (IQR)	7.3 (5.3, 10.5)	7.1 (4.9, 9.6)	7.4 (5.6, 10.0)	
FFMI				**<0.001 ^1^**
Mean (SD)	22.5 (1.8)	18.5 (1.5)	18.2 (1.7)	
Median (IQR)	22.1 (21.2, 23.5)	18.6 (17.5, 19.6)	18.4 (16.9, 19.6)	
SMI				**<0.001 ^1^**
Mean (SD)	11.0 (1.5)	8.4 (1.0)	8.5 (1.4)	
Median (IQR)	10.8 (10.1, 11.6)	8.4 (7.5, 9.2)	8.3 (7.3, 9.5)	
ASMI				**<0.001 ^1^**
Mean (SD)	8.8 (0.9)	7.0 (0.6)	6.6 (0.9)	
Median (IQR)	8.6 (8.2, 9.2)	7.0 (6.5, 7.4)	6.6 (6.0, 7.2)	
ECW-ICW ratio				**<0.001 ^1^**
Mean (SD)	0.8 (0.1)	0.9 (0.1)	1.3 (0.2)	
Median (IQR)	0.8 (0.7, 0.9)	0.9 (0.8, 1.0)	1.2 (1.1, 1.4)	

* *p*-values refer to Benjamini–Hochberg correction for multiple test comparisons; ^1^ Kruskal–Wallis rank sum test; and ^2^ Pearson’s Chi-squared test. Significant *p*-values are shown in bold. NRS-2002, Nutritional Risk Screening 2002; SARC-F, Strength, Assistance with walking, Rising from a chair, Climbing stairs, and Falls questionnaire; PhA, Phase Angle; BCMI, Body Cell Mass Index; FMI, Fat Mass Index; FFMI, Fat-free Mass Index; SMI, Skeletal Muscle Index; ASMI, Appendicular Skeletal Muscle Index; and ECW-ICW ratio, Extracellular Water–Intracellular Water ratio.

**Table 3:** Multivariable stepwise logistic analysis results for sarcopenia risk (SARC-F ≥ 4) (*n* = 832).

Characteristic	*n*	OR ^1^	95% CI ^1,2^	*p*-Value
Age (for each 5-years increase)	832	1.17	1.10, 1.25	**<0.001**
Neoplasm				**<0.001**
Head and neck	43	1.00	0.52 to 1.93	
Breast	91	2.59	1.63 to 4.12	
Digestive/gastrointestinal	311	2.71	2.12 to 3.46	
Genitourinary	195	1.17	0.83 to 1.65	
Gynecological	75	4.97	3.16 to 7.82	
Lung	117	11.0	8.31 to 14.51	
Education				**<0.001**
Less than 1st grade	136	1.00	0.75 to 1.33	
Middle school	269	0.53	0.42 to 0.66	
High school	299	0.38	0.31 to 0.48	
Graduated	128	0.32	0.22 to 0.47	
Diabetes				**0.001**
No	696	—	—	
Yes	136	1.70	1.23, 2.36	
Dyslipidemia				**0.041**
No	791	—	—	
Yes	41	1.76	1.02, 3.04	
Cancer surgery				**0.012**
No	698	—	—	
Yes	134	0.63	0.44, 0.90	
Other comorbidities				**0.002**
None	626	1.00	0.85 to 1.18	
One	173	1.60	1.25 to 2.06	
More	33	1.84	1.06 to 3.19	
NRS-2002				**<0.001**
<3	715	—	—	
≥3	117	4.81	3.38, 6.93	
Cluster				**0.006**
HMP	234	1.00	0.77 to 1.3	
MMP	389	1.01	0.83 to 1.22	
LMP	209	1.62	1.26 to 2.07	

^1^ OR = odds ratio; CI = confidence interval; and ^2^ CIs obtained with quasi-variance method of floating absolute risks. NRS-2002, Nutritional Risk Screening 2002; HMP, High Muscle Profile; MMP, Moderate Muscle Profile; and LMP, Low Muscle Profile. Significant *p*-values are shown in bold.

## Data Availability

The original data presented in this study are openly available from Zenodo at https://doi.org/10.5281/zenodo.15055450.

## Supplementary Materials

The following supporting information can be downloaded at https://www.mdpi.com/article/10.3390/nu17081376/s1, Supplementary Table S1: Distribution of BMI (numbers and class) according to age (≤65, >65 years); Supplementary Table S2: Distribution of BMI (numbers and class) according to gender; Supplementary Table S3: Distribution of BMI (numbers and class) according to cancer site; Supplementary Figure S1: Distributions of BIA-derived body composition measures and overall QoL Summary Score (SumSc); Supplementary Figure S2: Elbow plot representing the number of clusters in the k-means algorithm; Supplementary Table S4: Univariable analysis according to SumSc by median split; and Supplementary Table S5: Multivariable logistic analysis for sarcopenia risk (SARC-F ≥ 4) by median split of SumSc.

## Abbreviations

The following abbreviations are used in this manuscript:

BC	Body composition
MS	Muscle strength
QoL	Quality of Life
MM	Muscle mass
SMM	Skeletal muscle mass
FM	Fat mass
BIA	Bioelectrical Impedance Analysis
DXA	Dual-Energy X-Ray Absorptiometry
CT	Computed Tomography
MRI	Magnetic Resonance Imaging
PhA	Phase Angle
TBW	Total Body Water
BCM	Body Cell Mass
ECW	Extracellular Water
ICW	Intracellular Water
ML	Machine learning
PCA	Principal Component Analysis
MOGs	Multidisciplinary Oncology Groups
NRS-2002	Nutritional Risk Screening 2002
BMI	Body Mass Index
SARC-F	Strength, Assistance with walking, Rising from a chair, Climbing stairs, and Falls
WC	Waist circumference
HC	Hip circumference
FFM	Fat-free Mass
SMM	Skeletal muscle mass
ASMM	Appendicular skeletal muscle mass
FMI	Fat Mass Index
BCMI	Body Cell Mass Index
FFMI	Fat-free Mass Index
SMI	Skeletal Muscle Index
ASMI	Appendicular Skeletal Muscle Index
SumSc	C30 Summary Score
PCs	Principal Components
