# Supplementary material for: Optimizing Nutritional Care with Machine Learning: Identifying Sarcopenia Risk Through Body Composition Parameters in Cancer Patients—Insights from the NUTritional and Sarcopenia RIsk SCREENing Project (NUTRISCREEN)
Source: Nutrients. 2025 Apr 18;17(8):1376. doi: 10.3390/nu17081376 (PMC12030622; doi:10.3390/nu17081376)
Supplement: Supplementary file 1 [file nutrients-17-01376-s001.zip › nutrients-3570560-supplementary.pdf]

Supplementary Materials

Supplementary Table S1: Distribution of BMI (numbers and class) according to age (≤65,>65 years)

Characteristic	≤65, N = 504	>65, N = 470	p-value[1]
BMI (numbers)			0.42
Mean (SD)	27.43 (5.51)	27.06 (4.88)	
BMI, n (%)			0.25
<25	180 (35.7%)	176 (37.4%)	
25-30	182 (36.1%)	188 (40.0%)	
≥30	142 (28.2%)	106 (22.6%)	

[1]Benjamini & Hochberg correction for multiple testing. BMI, Body Mass Index

Supplementary Table S2: Distribution of BMI (numbers and class) according to gender.

Characteristic	Female, N = 430	Male, N = 544	p-value[1]
BMI			0.72
Mean (SD)	27.22 (5.47)	27.28 (5.00)	
BMI_cat, n (%)			0.72
<25	158 (36.7%)	198 (36.4%)	
25-30	158 (36.7%)	212 (39.0%)	
≥30	114 (26.5%)	134 (24.6%)	

[1]Benjamini & Hochberg correction for multiple testing. BMI, Body Mass Index

Supplementary Table S3: Distribution of BMI (numbers and class) according to cancer site.

Variables	**H&N** N = 61	**Breast**, N = 98	**DG**, N = 346	**Genitourinary**, N = 210	**Gynecological**, N = 83	**Lung**, N = 121	**Skin**, N = 55	**p-value**[1]
**BMI**								**<0.001**
Mean (SD)	26.39 (6.28)	27.15 (4.94)	26.07 (4.32)	28.48 (4.96)	29.13 (6.94)	27.63 (5.70)	27.52 (4.57)	
**BMI_cat, n (%)**								**<0.001**
<25	25.00 (40.98)	34.00 (34.69)	155.00 (44.80)	51.00 (24.29)	28.00 (33.73)	46.00 (38.02)	17.00 (30.91)	
25-30	23.00 (37.70)	39.00 (39.80)	126.00 (36.42)	90.00 (42.86)	25.00 (30.12)	41.00 (33.88)	26.00 (47.27)	
≥30	13.00 (21.31)	25.00 (25.51)	65.00 (18.79)	69.00 (32.86)	30.00 (36.14)	34.00 (28.10)	12.00 (21.82)	

[1]Benjamini & Hochberg correction for multiple testing; BMI, Body Mass Index; H&N, Head and Neck; DG, Digestive/Gastrointestinal

Supplementary Figure S1: Distributions of BIA-derived body composition measures and overall QoL summary scores (SumSc).

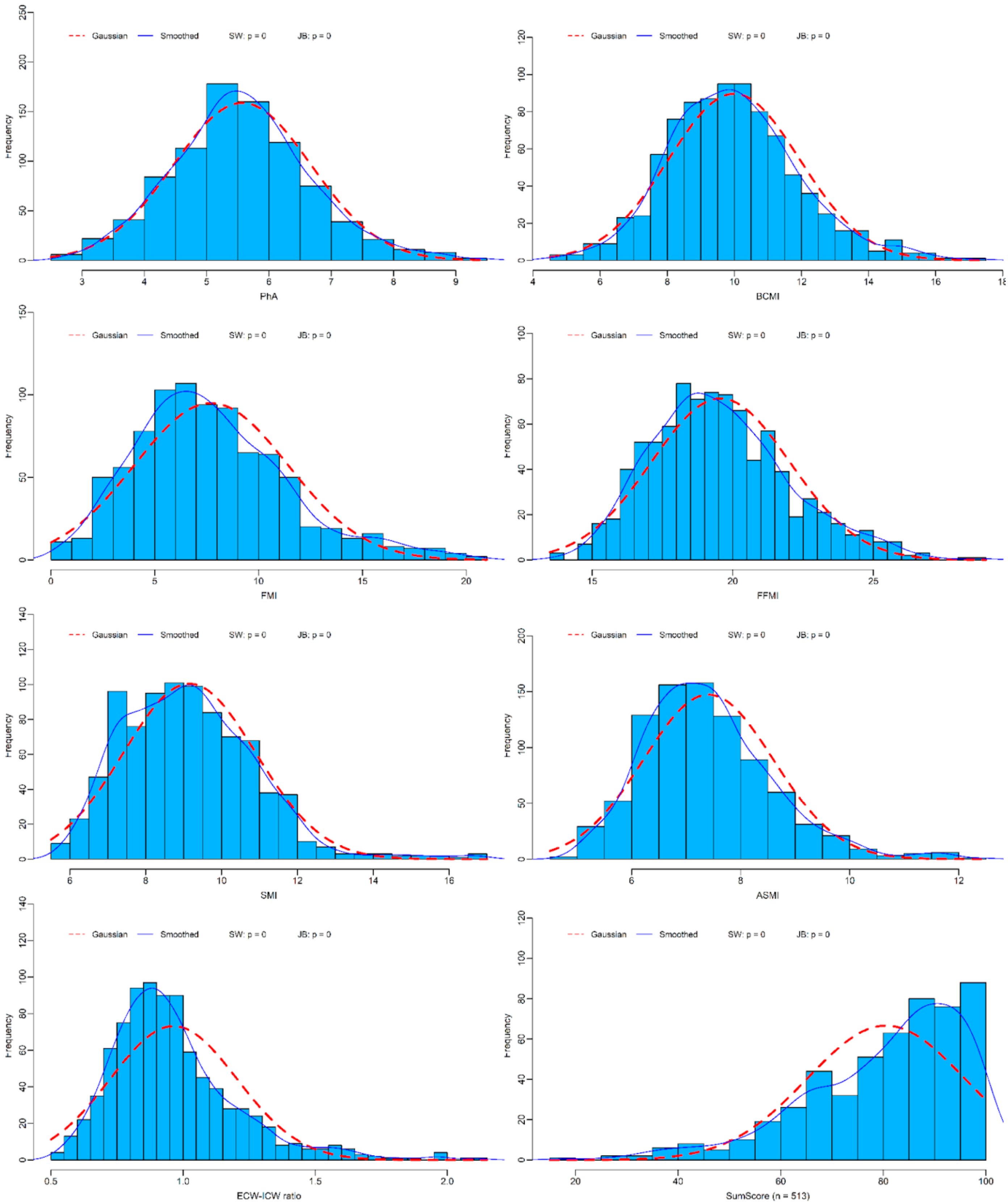

Supplementary Figure S2: Elbow Plot representing the number of clusters in k-means algorithm

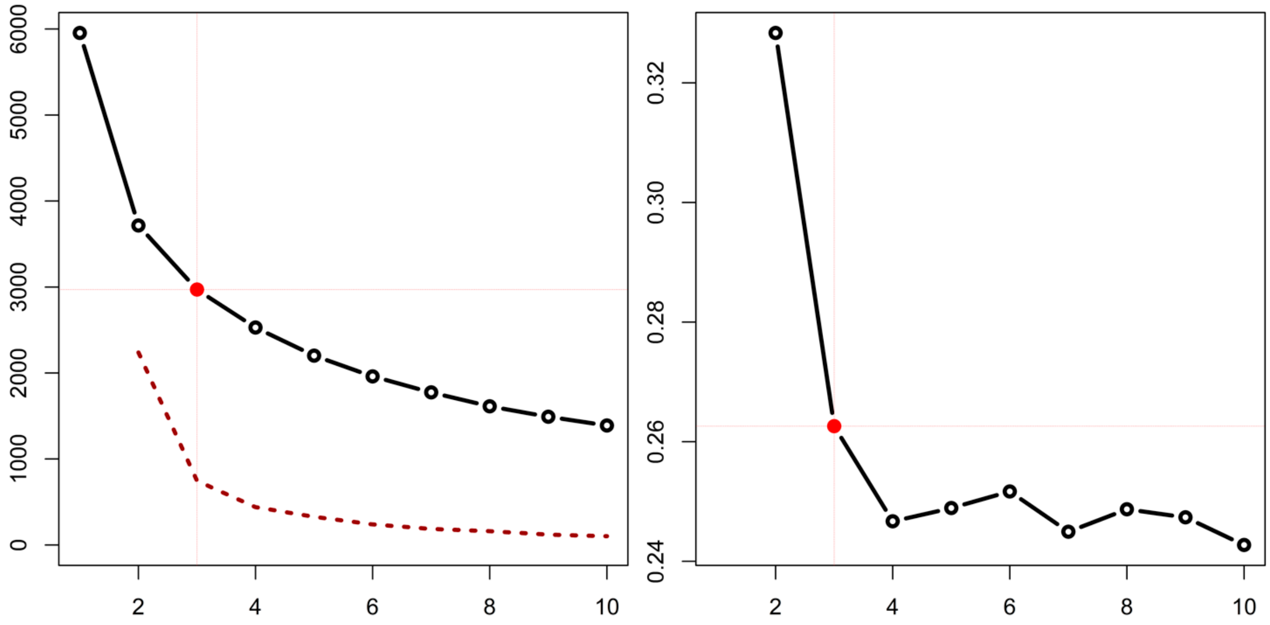

Elbow plot (left side) and mean silhouette score (right side) to determine the number of cluster in k-means algorithm. On the y-axis of the Elbow plot is visible the within-cluster sum of squares (sum of the distances between points from the same cluster centroid, WCSS): the more distances decrease, the sample is better partitioned, but having a large set of clusters is useless. Elbow method is an heuristic method aiming to find the best trade-off between good clusterization and number of groups, and it should be respected when WCSS starts decreasing in a linear trend. We detected as three the optimal number of clusters, as the difference of WCSSs from the k-means on three groups and the k-means on two is quite similar than the one calculated as the difference of WCSSs from the k-means on four groups and the k-means on three (dotted line). The silhouette score is the mean value of datapoints of a single cluster representing how similar they are each others. High values indicate that the data composing that cluster are meanly well matched to their own cluster. The mean score is the mean across the selected clusters.

Supplementary Table S4: Univariable analysis according to SumSC by median split.

	SumSC (n = 468)		
**Variable**	**SumSC ≤84, N = 235 (50%)**	**SumSC >84, N = 233 (50%)**	**p-value[1]**
Gender, n (%)			0.014[1]
Female	114 (48.5%)	85 (36.5%)	
Male	121 (51.5%)	148 (63.5%)	
Age (ys)			0.610[2]
Mean (SD)	63.8 (12.5)	63.7 (10.6)	
Median (IQR)	65.5 (54.7, 74.3)	64.6 (57.1, 71.9)	
Neoplasm, n (%)			0.002[1]
Head and neck	21 (8.9%)	10 (4.3%)	
Breast	27 (11.5%)	30 (12.9%)	
Digestive/gastrointestinal	66 (28.1%)	47 (20.2%)	
Genitourinary	47 (20.0%)	87 (37.3%)	
Gynecological	25 (10.6%)	25 (10.7%)	
Lung	49 (20.9%)	34 (14.6%)	
Education, n (%)			0.008[1]
Less than 1st grade	42 (17.9%)	20 (8.6%)	
Middle school	82 (34.9%)	69 (29.6%)	
High school	81 (34.5%)	97 (41.6%)	
Graduated	30 (12.8%)	47 (20.2%)	
Smoking status, n (%)			0.089[1]
No smoking	53 (22.6%)	75 (32.2%)	
Former smoker	110 (46.8%)	99 (42.5%)	
Smoker	72 (30.6%)	59 (25.3%)	
Hypertension, n (%)			0.139[1]
No	131 (55.7%)	113 (48.5%)	
Yes	104 (44.3%)	120 (51.5%)	

Diabetes, n (%)			0.011[1]
No	189 (80.4%)	209 (89.7%)	
Yes	46 (19.6%)	24 (10.3%)	
Cardiovascular disease, n (%)			0.008[1]
No	203 (86.4%)	220 (94.4%)	
Yes	32 (13.6%)	13 (5.6%)	
Dyslipidemia, n (%)			0.660[1]
No	224 (95.3%)	220 (94.4%)	
Yes	11 (4.7%)	13 (5.6%)	
Hypercholesterolemia, n (%)			0.100[1]
No	187 (79.6%)	169 (72.5%)	
Yes	48 (20.4%)	64 (27.5%)	
Cancer surgery, n (%)			0.130[1]
No	192 (81.7%)	203 (87.1%)	
Yes	43 (18.3%)	30 (12.9%)	
Other comorbidities, n (%)			<0.001[1]
None	155 (66.0%)	196 (84.1%)	
One	65 (27.7%)	33 (14.2%)	
More	15 (6.4%)	4 (1.7%)	
NRS-2002, n (%)			<0.001[1]
<3	184 (78.3%)	225 (96.6%)	
≥3	51 (21.7%)	8 (3.4%)	
SARC-F, n (%)			<0.001[1]
<4	177 (75.3%)	221 (94.8%)	
≥4	58 (24.7%)	12 (5.2%)	
Cluster, n (%)			0.014[1]
HMP	50 (21.3%)	71 (30.5%)	
MMP	110 (46.8%)	114 (48.9%)	

LMP		75 (31.9%)	48 (20.6%)

[1,2]P-values refer to Benjamini-Hochberg correction for multiple tests comparisons; [1]Pearson's Chi-squared test; [2]Kruskal-Wallis rank sum test. NRS-2002, Nutritional Risk Screening 2002; SARC-F, SARC-F, Strength, Assistance with walking, Rise from a chair, Climb stairs and Falls; HMP, High Muscle Profile; MMP, Moderate Muscle Profile; LMP, Low Muscle Profile.

Supplementary Table S5: Multivariable logistic analysis for sarcopenia risk (SARC-F ≥4) by median split of SumSC.

	**SumSC ≤84 (n = 235)**			**SumSC >84 (n = 233)**		
**Characteristic**	**N**	**OR (95% CI)[1,2]**	**p-value**	**N**	**OR (95% CI)[1,2]**	**p-value**
Age (ys)	235	1.20 (1.06 to 1.36)	0.003	233	1.21 (1.04 to 1.42)	0.015
Neoplasm[3]			<0.001			
Head and neck	21	1 (0.35 to 2.87)				
Breast	27	5.57 (2.56 to 12.1)				
Digestive/gastrointestinal	66	10.3 (6.36 to 16.5)				
Genitourinary	47	2.54 (1.34 to 4.81)				
Gynecological	25	7.21 (3.32 to 15.7)				
Lung	49	30.0 (19.0 to 47.4)				
Education			0.005			0.068
Less than 1st grade	42	1 (0.59 to 1.69)		20	1 (0.33 to 3.03)	
Middle school	82	0.95 (0.66 to 1.37)		69	0.84 (0.42 to 1.68)	
High school	81	0.41 (0.27 to 0.62)		97	2.24 (1.42 to 3.51)	
Graduated	30	0.41 (0.19 to 0.91)		47	2.18 (1.12 to 4.25)	
Diabetes[3]			0.161			
No	189	1				
Yes	46	1.50 (0.85 to 2.71)				
Dyslipidemia[3]			0.134			
No	224	1				
Yes	11	2.17 (0.79 to 6.40)				
Cancer surgery			0.091			0.285
No	192	1		203	1	
Yes	43	0.58 (0.31 to 1.09)		30	0.57 (0.18 to 1.56)	

Other comorbidities[4]			0.353			<0.001
None	155	1 (0.75 to 1.33)		196	1	
One	65	0.85 (0.55 to 1.32)		37	3.61 (1.73 to 7.56)	
More	15	1.67 (0.73 to 3.84)				
NRS-2002			0.004			0.009
<3	184	1		225	1	
≥3	51	2.34 (1.30 to 4.32)		8	4.89 (1.50 to 17.0)	
Cluster			0.354			0.008
HMP	50	1 (0.59 to 1.71)		71	1 (0.55 to 1.83)	
MMP	110	1.20 (0.86 to 1.68)		114	0.78 (0.47 to 1.29)	
LMP	75	1.62 (1.05 to 2.50)		48	2.53 (1.43 to 4.47)	

[1]OR = Odds Ratio, CI = Confidence Interval; [2]CIs obtained with quasi-variance method of floating absolute risks; [3]variables not entered in the model for Summary score >84: no patients with diabetes or dyslipidemia conditions or with more comorbidities had a SumSc >84; [4]the OR in the model for Summary score >84 is referred to the presence of comorbidities (“one or more”). NRS-2002, Nutritional Risk Screening 2002; HMP, High Muscle Profile; MMP, Moderate Muscle Profile; LMP, Low Muscle Profile.
